# Increasing Authenticity of the Laboratory through the MICRO Project: Analysis of Analytical Chemistry Laboratory Experiments for Their Level of Inquiry

**DOI:** 10.1021/acs.jchemed.3c00945

**Published:** 2024-12-17

**Authors:** Andrea L. Van Wyk, Kimberley A. Frederick, Marya Lieberman, Renée S. Cole

**Affiliations:** † Department of Chemistry, University of Iowa, Iowa City, Iowa 52242, United States; ‡ Department of Chemistry, Skidmore College, Saratoga Springs, New York 12866, United States; § Department of Chemistry and Biochemistry, University of Notre Dame, Notre Dame, Indiana 46556, United States

**Keywords:** Second-Year Undergraduate, Laboratory Instruction, Inquiry-Based/Discovery Learning, Professional Development, Chemistry Education Research

## Abstract

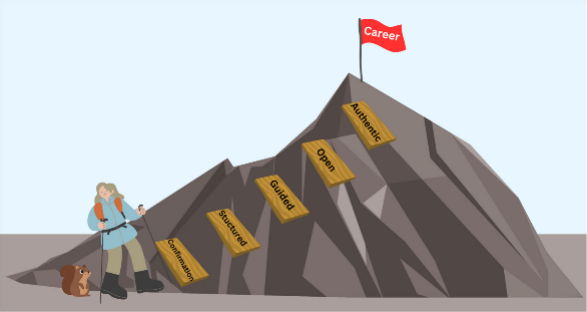

Inquiry-based laboratory experiments, in comparison to traditional “cookbook” style laboratory experiments, more accurately model the work scientists do and engage students in the skills they use. Students who participate in inquiry-based laboratory experiments engage in science practices and develop skills such as critical thinking and argumentation. Despite the abundance of literature surrounding the benefits of inquiry-based laboratory learning approaches, adoption of these instructional approaches has been slow. The MICRO project was designed with the goal of supporting analytical chemistry faculty in adopting inquiry-based laboratory experiments. Laboratory experiments representing those used by institutions prior to the project and during the project were collected and analyzed for their level of inquiry. We saw a decrease in the percentage of laboratory experiments that were traditional, “cookbook” style laboratory experiments and an increase in laboratory experiments that are higher levels of inquiry during the semester of implementation of MICRO laboratory experiments, indicating faculty were adopting some inquiry-based instructional practices.

## Introduction

Over the past decade a lot of attention in science education has focused on preparing students to be scientists through cultivating necessary skills and practices.^1,2^ Many studies have highlighted a skills gap where students leaving college are not equipped with the necessary skills that employers desire.^3−6^ Because of this, *A Framework for K-12 Science Education* and the Next Generation Science Standards for K-12 education emphasized science and engineering practices, summarized in a list of eight practices that are integral to the work of scientists and engineers.^1,2^ Higher education is starting to follow suit by finding ways to intentionally integrate these necessary skills and practices into programs of study. For chemistry curricula in particular, one avenue for this integration is in laboratory learning.^7,8^

### A Call for Reforming Laboratory Learning

Learning in the laboratory has been a point of discussion among chemists and educators throughout the history of chemistry education. Many argue that the laboratory is a vital part of a chemist’s education because it is the place where students “do chemistry” and can see chemistry come to life in ways a lecture course may fall short.^9−11^ For these same reasons professional bodies such as the American Chemical Society Committee on Professional Training mandate at least 350 h of laboratory experience beyond the introductory sequence for bachelor degree programs.^12,13^ However, others have drawn attention to the lack of rigorous research studies that provide evidence of the vital role the laboratory plays in chemistry degree training despite the long history of laboratory learning.^10,14^ With more universities questioning the role of laboratory learning, there is a renewed interest within the chemistry education community for research on the role and nature of laboratory work within chemistry curriculum and any skills that may be unique to laboratory courses.^10,11^ Leaders have pointed to the potential of laboratory courses to be a place in the chemistry degree where students learn *how* to do science, rather than just *doing* science.^11^ In doing so students will gain the necessary skills and practices that are integral to scientists.^1^ The key to doing so is to move away from “cookbook” laboratory experiments toward instructional approaches, such as inquiry-based laboratory experiments, that model more authentic research.^10^

### Benefits of Inquiry-Based Laboratory Learning

Many chemistry laboratory courses predominantly have students engage in traditional “cookbook” style laboratory experiments. These types of laboratory experiments, as their name implies, provide students with the research question, the necessary background information, and step-by-step directions to collect and analyze the data to draw a final conclusion to the question at hand.^15^ ”Cookbook” style laboratory experiments primarily require that students can follow directions well. While that is a skill that instructors or future employers may want to cultivate in students, these types of laboratory experiments severely limit the opportunities students have to engage in inquiry. Since inquiry is a term that is somewhat ill-defined within the science education community,^16^ for the purposes of this work, we are referring to inquiry in the sense of inquiry-based instruction as put forth by French and Russell:

Inquiry-based instruction places more emphasis on the students as scientists. It places the responsibility on the students to pose hypotheses, design experiments, make predictions, choose the independent and dependent variables, decide how to analyze the results, identify underlying assumptions, and so on. Students are expected to communicate their results and support their own conclusions with the data they collected.^17^

Inquiry-based laboratory experiments contrast with “cookbook” laboratory experiments in terms of what is provided to the students (teacher-directed) and what decisions the students make while completing the experiment (student-directed). Inquiry-based laboratory experiments provide students opportunities to make key decisions throughout the experiment and are more open-ended and less prescriptive in nature so that students can engage in problem solving, experimental planning, and evaluating data. However, inquiry-based laboratory experiments can vary in their degree of inquiry, with lower inquiry laboratory experiments being more teacher-directed and higher inquiry laboratory experiments being more student-directed.^15^ Guided inquiry experiments, as defined by Bruck et al., are a step higher in inquiry than cookbook laboratory experiments in that they provided students the opportunity to evaluate their data and draw conclusions while still providing complete background and procedural information.^15^ These laboratory experiments can be useful in starting to transition students toward inquiry-based laboratory experiments or for introducing new procedural techniques or instruments. Higher levels of inquiry, such as authentic inquiry, include project-based laboratory experiments where students must determine a research question of interest, determine necessary background information, and then design (and maybe redesign) an experimental plan to collect and analyze data to answer that research question.^15^ This variety in levels of inquiry can be useful for scaffolding student experiences to progress to greater levels of independence and engagement in inquiry throughout a course and throughout a student’s progression in a degree program.

Within the chemistry education community, a number of different inquiry-based laboratory approaches have been studied to determine the impact of inquiry-based laboratory education on students’ laboratory learning experiences.^18−21^ Studies investigating student learning experiences with inquiry-based laboratory experiments have shown increases in practical exam scores,^22^ improved critical thinking,^23^ increased self-reflection of learning,^19^ closing of performance gaps,^18,20,22^ increased proficiency with developing arguments,^24,25^ and a more complex understanding of the phenomenon of study.^19^ Another benefit of inquiry-based laboratory learning is the opportunities for engaging in science practices.^22,26,27^ In a study by Carmel et al. a traditional General Chemistry laboratory curriculum and a project, inquiry-based laboratory curriculum were analyzed for their opportunities to engage students in the eight Scientific and Engineering Practices^28,29^ using aspects of the 3D-Learning Assessment Protocol.^28,30^ They found that the project based laboratory curriculum provided significantly more opportunities for engagement in science practices than the traditional laboratory curriculum.^28^ These are vital opportunities for students to develop the skills necessary to be working scientists.

### Lack of Adoption of Inquiry-Based Laboratory Instruction

While the benefits of inquiry-based laboratory activities have been demonstrated consistently over the years,^18−20,22−25^ cookbook laboratory activities still dominate the curriculum in many chemistry laboratory courses. In an analysis of laboratory experiments in STEM done by Bruck et al. it was found that only 8% of the 386 laboratory experiments analyzed were inquiry-based.^15^ Additionally, investigating just chemistry laboratory experiments they found that only 11% of the 229 laboratory experiments analyzed were inquiry-based.^31^ In addition, two recent large national surveys have shown that faculty still self-report heavily relying on cookbook style laboratory experiments in their laboratory courses.^32,33^ Especially relevant to this study, one national survey focused on inorganic chemistry laboratory courses, which are often completed later in the chemistry major sequence, where we may anticipate seeing a greater amount of higher-inquiry laboratory experiments.^33^ This lack of widespread adoption is especially concerning as there have been calls put forth for more inquiry-based learning opportunities in order to equip students with the necessary skills valued postgraduation.^34^

Changing faculty instructional practices is no easy task due to the numerous factors impacting those practices and the complex system faculty are immersed in. Typical approaches to encourage faculty to adopt new instructional approaches, such as the “documentation and dissemination” approach, rarely prove successful.^35^ Some commonly cited barriers for adoption include a lack of time, money, and proper training.^36^ Another common barrier to adoption is faculty beliefs. Studies have found alignment between chemistry faculty’s beliefs and the instructional approaches they use in their courses.^37,38^ Change literature also demonstrates that before an instructor will change their practice, they have to have a desire to change, or a belief that another approach will work better.^35^ Additionally institutional barriers such as content-coverage expectations, class size, and room layout can often impede adoption of such instructional approaches.^39^ Along with these barriers, change literature has also identified three drivers for long-term adoption of instructional practices: 1) adequate time to make the change, 2) reflection on the change, and 3) receiving feedback on the change.^35,40,41^ With these barriers and drivers for adoption in mind, we started the MICRO project^42^ to equip and empower analytical chemistry instructors to adopt inquiry-based instructional practices.

## Theoretical Framework

The theoretical frameworks guiding this research study are situated learning and constructive alignment. Situated learning is a learning theory originating from the works of Brown et al.^43^ and Lave and Wenger^44−46^ that describes learning as situated in a specific context and social environment. Theorists with this lens emphasize that learning is a generative process where knowledge is constructed through an individual’s interaction with the context and other individuals in the environment. Because of this, an individual’s understanding of a concept is constantly under construction.^47^ Within this model Lave and Wenger define learning as the process of increased participation within a community of practice (CoP).^45^ A CoP is a group of individuals who share common goals, beliefs, and norms of practices.^45^ When a newcomer joins a CoP they are on the periphery of the CoP. As they start to participate and interact with other members of the community, they learn about goals, techniques, and language of the community, becoming more proficient and invested within it.

Students taking chemistry courses, particularly those working on a degree in chemistry, are integrating into a CoP that is the discipline of chemistry itself. In the case of our study, we are investigating the level of inquiry of laboratory experiences students will engage with. In terms of situated learning, the goal is for students to become equipped to engage in authentic practices of a chemist. Authentic work of chemists requires individuals to make decisions and employ skills such as problem solving. For a novice to integrate into the community and move toward becoming an expert, they need the opportunity to engage in more authentic practices.

The framework of constructive alignment, which consists of three elements - intended learning objectives, tasks, and assessment, also guided this work. Constructive alignment serves as a model for creating or modifying curricula to align with the intended learning outcomes, ensuring that students have opportunities to practice and be assessed on those learning outcomes.^48,49^ For this work we define the intended learning objectives as engaging in authentic work of scientists, specifically scientific inquiry. The tasks we are investigating are the laboratory activities that students complete, including their level of inquiry. Lastly the assessments are the deliverables for the laboratory activity, often a laboratory report or presentation. These assessments should include explicit prompts for students to demonstrate their proficiency with these skills. In our study we are focusing on the tasks element of constructive alignment to investigate the extent to which they are designed to be aligned with the goal of engaging students in scientific inquiry.

## The MICRO Project Aimed to Support the Adoption of Inquiry-Based Laboratory Experiments

Experts in the MICRO project^42^ designed a suite of eight analytical chemistry laboratory experiments that use paper-based microfluidic technology and tiny amounts of reagents to safely run traditional experiments at home as well as in the laboratory. The MICRO laboratory experiments covered many of the topic areas and laboratory techniques in a typical sophomore analytical chemistry course such as acid–base titrations, calibration curves, standard addition, and electrochemistry.^42,50,51^ In addition to these content goals, the MICRO laboratory experiments were also intentionally designed to be inquiry-based and provide students opportunities to engage in science practices.^42,51^ More information on the MICRO project and MICRO experiments can be found in Roller et al.^42^

The overall structure of the MICRO project was designed to alleviate some of the known barriers for faculty adoption of evidence-based instructional practices (EBIPs). One approach was providing ready to implement and low-cost laboratory experiments on traditional analytical chemistry topics to reduce the time to design new activities and the money needed to equip a laboratory for a new experiment. We asked participating instructors to implement two to four inquiry-based laboratory experiments throughout a semester to allow them ample opportunities to interact with the inquiry nature of the laboratory experiments, but still have the comfort and convenience of using previous laboratory experiments. We developed an online 3-day workshop to introduce faculty to the microfluidic technology, the nature of inquiry-based laboratory experiments, and facilitation strategies necessary for guiding students through an inquiry-based laboratory experiment. We also designed the MICRO project to encourage faculty to reflect upon their changes and provided feedback to participants about the levels of inquiry observed in their laboratory experiments.^35^

Since studies show that being involved in a community of practice can help with the adoption of EBIPs,^52−55^ the MICRO project established a structure to support participants while they adopted inquiry-based learning practices. This included an online platform, Trello, which allowed participants and MICRO team members to pose questions and share resources. The MICRO team members also frequently hosted office hours during the semesters when participants were implementing laboratory experiments. Many participants were active in the community of practice and engaged frequently with these resources. To better understand the impact of involvement in the MICRO workshops and community on faculty’s instructional practices in the laboratory we sought to answer the following research question: What are the levels of inquiry of laboratory experiments used by participants in the MICRO project before and during their involvement in the project?

## Methods

### Data Collection

We recruited applicants for the MICRO project by advertising through established email lists, online communities, and social media. After receiving applications, we invited about 20 participants for each cohort (cohort 1: 2020–2021, cohort 2: 2021–2022) to join the project. In doing so they completed the National Survey of Faculty Goals (NSFG) survey and a questionnaire about their views on the purpose of laboratory learning in their analytical chemistry laboratory course.^56^ Participants also submitted representative examples (the case for cohort 1 participants) or their whole semester (the case for cohort 2 participants) of the handouts of the laboratory experiments they used the last time they taught their analytical chemistry laboratory course. At the beginning of August we held a 3-day, online faculty development training where participants could put themselves in their students’ shoes and try some of the MICRO experiments (materials were sent to them in advance) as well as learn how to write well-designed learning objectives and learn about facilitating and assessing inquiry-based laboratory experiments. For more information about the faculty development training see Roller et al.^42^

During the fall and/or spring semester, participants implemented at least two of the MICRO laboratory experiments in their analytical chemistry course. The MICRO team provided support mechanisms for implementing these laboratory experiments by providing virtual “open hours” where participants could come ask the team and other participants questions as well as an online Trello board where shared resources could be posted.^42^ At the conclusion of their semester of involvement, participants completed the NSFG survey and a questionnaire similar to what they completed at the start of the project. Participants also submitted all the laboratory experiments their students completed during the semester, including the MICRO laboratory handouts they used. For this study we examined the laboratory materials that faculty submitted that represent their instructional practices both prior to and during their engagement with the MICRO project.

For our study we analyzed materials from two cohorts of faculty who were involved in the MICRO project. Cohort 1 consisted of 20 faculty from 19 institutions during the 2020–2021 academic year. Two faculty from one institution team-taught an analytical course and implemented the MICRO laboratory experiments together. Of those 19 participating institutions, 16 (84.2%) consented to having their data used for this study. Of those 16 participating institutions, 13 (68.4%) submitted a complete data set of laboratory experiments for analysis. This cohort implemented while the COVID pandemic was still impacting the modality to which institutions were having classes: faculty from 4 institutions taught completely remote, 4 institutions taught in a variation of a hybrid course, and 5 institutions taught completely in-person. Cohort 2 consisted of 19 faculty from 18 institutions during the 2021–2022 academic year, with two faculty team-teaching a course. Of those 18 participating institutions, 9 (50%) institutions consented to having their data used for this study. Of those 9 participating institutions, 4 (22.2%) submitted a complete data set of laboratory experiments for analysis. At this time, more institutions were back to their original modality of learning, and the faculty at the 4 institutions were teaching fully in-person. Faculty in both cohorts taught at a variety of institutions, ranging from baccalaureate colleges to doctoral universities. The breakdown of institution types (Carnegie classification) is provided in Table 1.

**Table 1:** Summary of Participating Institutions Whose Data Was Included in the Study

Cohort	Baccalaureate College	Masters College	Doctoral University
Cohort 1	6 (46.2%)	5 (38.5%)	2 (15.4%)
Cohort 2	1 (25%)	2 (50%)	1 (25%)

### Modifications of the Levels of Inquiry Rubric

The level of inquiry of laboratory experiments submitted by faculty in the MICRO project, both before and during their semester(s) of involvement in the project, was analyzed using a modified version of the inquiry rubric designed by Bruck, Bretz, and Towns^15,31^ that defined levels of inquiry by the amount of structure in laboratory experiments provided to students. Traditional “cookbook” style laboratory experiments are defined as lower levels of inquiry, where the characteristics of the laboratory experiments are provided to the students, while the higher levels of inquiry laboratory experiments do not provide as many of the laboratory characteristics to the students. The original elements of this rubric can be seen shaded in yellow below in Table 2.

**Table 2:** Modified Levels of Inquiry Rubric Based on the Rubric by Bruck et al. (2008); Original Levels Included in the Bruck, Bretz, Towns 2008 Rubric Are Indicated in Bold Font^15^

Characteristic	**Level 0: Confirmation**	**Level 0.5: Structured Inquiry**	**Level 1: Guided Inquiry**	Level 1 Alternative	Level 1.5	**Level 2: Open Inquiry**	Level 2 Alternative	Level 2.5	**Level 3: Authentic Inquiry**
Problem/Question	**Provided**	**Provided**	**Provided**	Provided	Provided	**Provided**	Provided	Provided	**Not provided**
Theory/Background	**Provided**	**Provided**	**Provided**	Provided	Provided	**Provided**	Partially provided	Not provided	**Not provided**
Procedure/Design	**Provided**	**Provided**	**Provided**	Partially provided	Half provided	**Not provided**	Partially provided	Not provided	**Not provided**
Results analysis	**Provided**	**Provided**	**Not provided**	Partially provided	Not provided	**Not provided**	Not provided	Not provided	**Not provided**
Results communication	**Provided/Partially provided**	**Provided/Partially provided**	**Partially provided/Not provided**	Partially provided/Not provided	Partially provided/Not provided	**Partially provided/Not provided**	Partially provided/Not provided	Partially provided/Not provided	**Partially provided/Not provided**
Conclusions	**Provided**	**Not provided**	**Not provided**	Not provided	Not provided	**Not provided**	Not provided	Not provided	**Not provided**

In applying the original rubric to the submitted laboratory experiments, we determined that it was difficult to characterize laboratory experiments that fell in between levels. For instance, if a laboratory experiment first provided prescribed steps for the students to collect data but then directed them to redesign the experiment to improve the process, neither open inquiry (level 2) nor guided inquiry (level 1) accurately characterizes this scenario since the procedures/design is not fully provided nor fully not provided to the students. We saw many laboratory experiments that had scenarios of some degree of partially provided laboratory characteristics and sought to quantitatively capture these laboratory experiments. We expanded Bruck et al.’s rubric to add some alternative levels as well as some half levels as seen in Table 2. It is also worth noting that we are not the first to modify this inquiry rubric to capture laboratory experiments that partially provide laboratory characteristics to students. Moss and Cervato modified the Bruck inquiry rubric in their study of the level of inquiry of reformed geology laboratory experiments so that each laboratory characteristic had three options: provided, transitional, and not provided.^57^ This addition of the transitional category is similar in nature to what we’ve defined as “partially provided”. We are defining “partially provided” as places where students must make decisions but are still provided with 50% or more of that characteristic. An example of “partially provided” procedure could be having students determine an appropriate dilution but then still providing students with the bulk of the experimental protocol. The addition of half levels is also not unique to our work, as Bruck et al. initially had half levels beyond level 0.5 but eliminated them in development since they did not have any laboratory experiments that fell into those categories, as well as other authors who have designated half levels in their application of this rubric.^31,57,58^ For the purposes of our work, we have defined “half provided” when a significant (typically about 50%) portion of the laboratory characteristic is left up to the student. One example is a two-week procedure where the first week students are given a protocol for an experiment and then the second week they have to redesign the experiment from the week prior to improve it or answer a new question.

The other difference in comparing the Bruck et al. rubric and the modified rubric we are using is the flexibility in defining the laboratory characteristic “results communication”.^15^ Bruck et al. defined “results communication” as that which “characterizes the manner by which data and experimental results are presented - are students given options on how to communicate results, or does the manual prescribe a specific method?”^15^ In examining laboratory experiments we discovered that the ways in which instructors directed students to communicate their findings of the laboratory experiment was variable. The type of artifact varied greatly in nature from traditional laboratory reports, presentations, and video reports to preprogrammed Excel sheets or simply just the reporting of a final numerical value. The other variable part of the results communication was the amount of support students were given in terms of *what* results to communicate. Some instructors wanted formal reports or presentations including figures and tables displaying data collected, some wanted supporting calculations, explanations, and interpretations of the data, and yet others wanted specific postlaboratory questions answered. Additionally, both *how* and *what* the students were instructed to communicate for their results were sometimes set as classroom norms, making it difficult to capture this characteristic fully for a single laboratory experiment. Because of these differences in both *what* needed to be communicated and *how* it needed to be communicated, we wanted our rubric to provide flexibility for the numerous scenarios that may be presented.

We defined 4 subcodes for results communication: provided, partially provided, not provided and N/A defined below in Table 3. For the lowest two levels of inquiry (level 0 and 0.5), which are the traditional “cookbook” style of laboratory experiments, we defined these levels to have either “provided” or “partially provided” results communication since these laboratory experiments are prescriptive in nature. For inquiry levels 1 (guided inquiry) and higher we defined these to have either “partially provided” or “not provided”. This flexibility in allowing “partially provided” allowed for scenarios where instructors may provide a few prompts or specifics about the format of the artifact but still provide significant opportunities for the student to make choices on *what* and/or *how* to present their findings. Because of this flexibility in subcodes for this characteristic, this laboratory component tends to not be a discriminating characteristic for level of inquiry and more significance is held in the other five laboratory characteristics.

**Table 3:** Subcodes for the “Results Communication” Component of Laboratory Experiments in Our Modified Levels of Inquiry Rubric

Subcode for Results Communication	Definition	Key Features
Provided	Students are restricted to reporting their results in a specified, provided and already organized fashion. Students do not decide what results to communicate and are not required to provide evidence or reasoning to support their conclusions in their artifact of communication.	Students are provided a formatted, organized prescribed method for communicating their results. Often this is in the format of an already organized spreadsheet, fill-in-the-blank laboratory report sheet, reporting a singular or list of values or answering laboratory postlab questions that are directive/specific and do not require students to craft arguments to support their claims.
Partially provided	Students are provided some guidance/prompting for how to communicate their findings but are given some opportunities for choices about what results to communicate and/or how to most effectively communicate their findings to the audience of interest.	Students are provided some scaffolding/guidance for what to include and the physical artifact for communicating their results, but students are given some places where they can make choices for how they want to communicate their findings to the audience of interest. This often comes in the form of students needing to identify what results would best communicate their findings or to support their answers with an argument or evidence. For laboratory reports this often is by providing students with what to generally include in the report but not what to use to support those claims. For postlab questions this is often providing questions that require an explanation and/or lets students decide what data is appropriate for supporting their claim.
Not provided	Students are given little to no direction on what data to present or how to report their findings. Formatting suggestions (such as include a background section or methods summary in your laboratory report) can be present and do not contribute to the overall degree to which the results communication is provided.	Students decide what data is appropriate to report to answer the research question and how best to organize their claims and arguments for the physical artifact where they are communicating their results. General guidance on what to include in a laboratory artifact does not impact the level. This often presents itself in project-based laboratory experiments or laboratory reports where a general outline is provided.
N/A	There is no component where students must communicate their findings.	

### Coding Process and Reliability Measures

As indicated above, the first and last authors expanded and refined the inquiry rubric to capture the various types of laboratory experiments present in the data set. This process was done iteratively until saturation was reached. Coding of laboratory experiments was done by examining each laboratory characteristic and assigning a subcode (provided, partially provided, not provided) to each characteristic. Then the overall level was determined by matching the list of subcodes to the level. From the 245 laboratory experiments submitted, 15% of laboratory experiments were used as a pilot set to establish initial reliability between the first and the last author. The first author then independently coded all the laboratory experiments. An additional 10% of laboratory experiments were co-coded with a second researcher throughout the duration of the coding to demonstrate continued reliability. A kappa value of κ = 0.631 was determined using Gwet’s AC1 statistic indicating moderate agreement.^59^ Despite improvements to the Bruck inquiry rubric^31^ to allow for laboratory experiments that fell in between discrete levels, the authors still had to make some judgment calls for laboratory experiments that fell between two levels, particularly between levels 0.5 and 1 due to varying levels of support provided to students with data analysis. Because of this, we report IRR for ±0.5 level as well, which yielded a kappa value of κ = 0.958, indicating almost perfect agreement.^60^

## Results and Discussion

### Levels of Inquiry of the MICRO Laboratory Experiments

The eight MICRO laboratory experiments that faculty could choose to implement in their course are listed in Table 4 along with their level of inquiry.^50^ These laboratory experiments were designed to range from levels 1–2 to move students beyond traditional “cookbook” style laboratory experiments (levels 0 and 0.5) but still provide some structure for using the microfluidic devices. Level 1.5 laboratory experiments, such as the Vinegar Titration MICRO laboratory experiment, had significant pieces of the experimental procedure that were left up to the student to figure out, such as the optimal location on the μpad for the end point to occur and the appropriate dilution factor. The level 2 laboratory experiment, Make Your Own Microfluidic Device, had students developing their own experimental plan to make and test a microfluidic device. Levels 1, 1.5, and 2 all required students to engage with data analysis, prompted by open-response postlaboratory questions. Faculty had access to student and instructor materials through the online community but were encouraged to modify the laboratory materials as needed to fit within their laboratory curriculum.^50^

**Table 4:** Levels of Inquiry of the Eight MICRO Laboratory Experiments, Ranging from Guided to Open Inquiry

MICRO Experiment	Level of Inquiry
Vinegar Titration	1.5
Ksp of Amphoteric Salts	1.5
Milk Protein Analysis	1.5
Bromide Water Analysis	1
Copper Electrochemistry	1
Make Your Own Microfluidic Device	2
Iodometric Vit C Titration	1.5
Iron Analysis of Vitamin Tablet	2

Since faculty at participating institutions were encouraged to make changes to the laboratory materials for the MICRO laboratory experiments, we analyzed the exact MICRO laboratory materials faculty used to make sure that any changes they made did not change the level of inquiry of the laboratory experiment. To do this, the student laboratory handouts collected from each institution were compared with the original student laboratory handout for the respective MICRO laboratory experiment and the deviations from the original materials were recorded and the level of inquiry was determined for the participant submitted MICRO laboratory experiments. We found that most institutions either used the materials exactly as provided or made minor changes that did not impact the level of inquiry of the laboratory experiments. Some of the small changes that institutions made included deleting or adding a learning objective, deleting or adding a prelab or postlab question, or changing the type of communication form from postlab questions to a report or presentation. There were a few isolated instances where the level of inquiry decreased when faculty added more detail to the procedure/design where it had initially been designed to be open-ended for students to make procedural decisions or the removal of prelab questions that had students making procedural decisions.

### Level of Inquiry—Prior to MICRO

**Figure 1:**
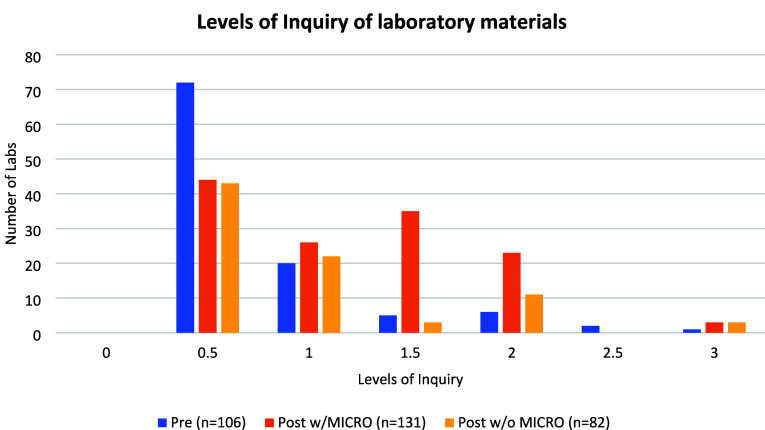
Results of the analysis of laboratory materials for their level of inquiry. Materials used by participants prior to being involved in the project are represented in blue, materials used during the semester of implementation including the MICRO laboratory experiments used are presented in orange, and the materials used during the semester of implementation excluding the MICRO laboratory experiments used are in yellow.

After applying the modified version of Bruck’s^15^ levels of inquiry rubric to the laboratory experiments submitted by institutions prior to their involvement in the MICRO project, we found that a majority (67.9%) of the laboratory experiments were structured inquiry (level 0.5) as seen in Figure 1.

Of the 17 institutions whose laboratory materials were analyzed, just under half (n = 8) had 1 or more laboratory experiments at a level 1.5 or higher. These findings align with those found by Bruck et al.^15,31^ in their analysis of laboratory experiments where a large majority of the laboratory experiments being used were confirmation (level 0) or structured inquiry (level 0.5), indicating a reliance on traditional, “cookbook” style laboratory experiments. This finding was not surprising as the nature of analytical chemistry courses often lends itself to prescriptive laboratory experiments, where the emphasis is on the precision/accuracy of techniques and not on cultivating a range of science practices. While there were a few institutions with one or two higher inquiry laboratory experiments, often final or group projects, the baseline for faculty entering the MICRO project was a heavy reliance on traditional laboratory experiments.

**Figure 2:**
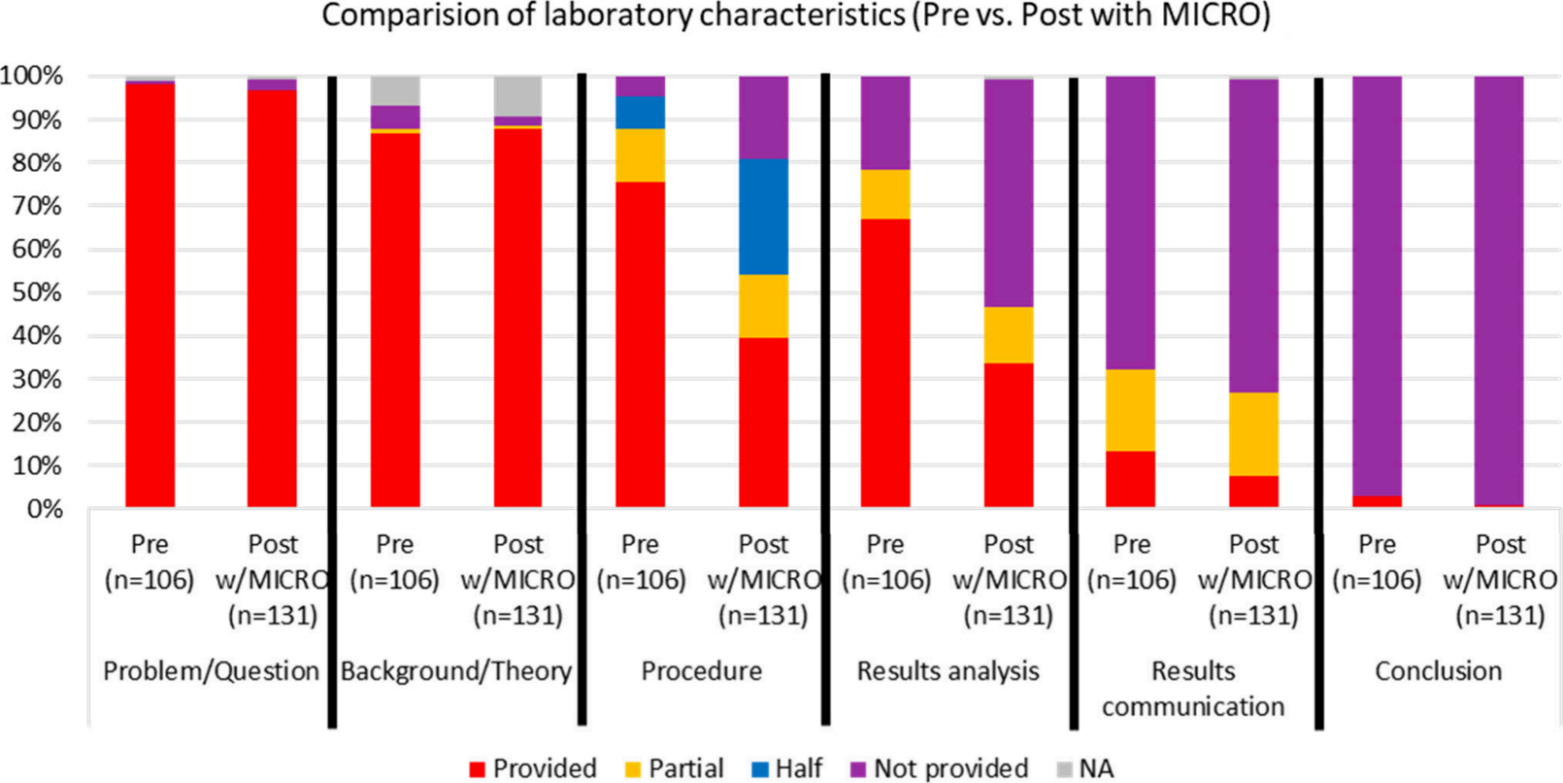
Results of the analysis of pre and post (with MICRO) laboratory materials broken down for each laboratory component.

Taking a closer look at the different laboratory components that were provided to students (or not provided to students), we can see in Figure 2 that for the preimplementation data set, a very high percentage of laboratory experiments provided students with the problem/question, background/theory, procedure, and results analysis. It was not until results communication and conclusion that we saw more opportunity for students to generate these aspects rather than having them provided, which aligned closely with level 0.5, structured inquiry laboratory experiments.

**Figure 3:**
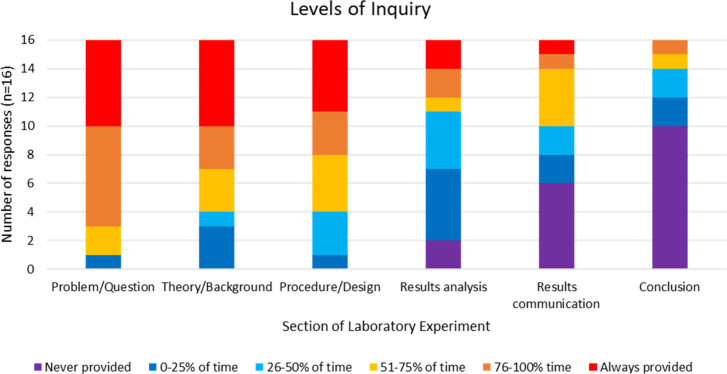
Presurvey results for faculty’s self-reported percentage of time laboratory characteristics were provided to students throughout their laboratory course.

This reliance on traditional, “cookbook” style laboratory experiments was aligned with the data self-reported by faculty in the survey they completed at the start of the MICRO project. Faculty were asked to select how often they provided the six laboratory characteristics (defined in Bruck et al.^15^) to students in laboratory experiments completed throughout the course. The data for faculty at 16 institutions (1 did not submit a presurvey) are reported in Figure 3.

We can see that most faculty reported frequently providing *problem/question, theory/background*, and *procedure/design*. At *results analysis* we saw more variation in responses, with a decrease in the percentage of time it was provided to students, indicating a guided inquiry laboratory experiment (level 1). At *results communication* and *conclusion* we see a decrease in the percentage of time these laboratory components are offered, indicating a structured inquiry (level 0.5) laboratory experiment. Comparing these self-reported results to the analysis of preimplementation laboratory materials we can see that faculty’s reflections on their laboratory materials were aligned with their actual practices, which were heavily “cookbook” style laboratory experiments. In two recent studies,^32,33^ a similar question was posed in a national study to inorganic chemistry faculty^33^ and faculty across all disciplines of chemistry^32^ where faculty reported the percentage of time the different laboratory components were generated by students in their laboratory experiments. Both studies’ findings were aligned with our findings indicating that faculty report using “cookbook” style laboratory experiments where students are provided with most of the laboratory components.

### Level of Inquiry—Semester of Implementation

After applying the modified version of Bruck’s^15^ levels of inquiry rubric to the laboratory experiments used by each institution during their involvement in the MICRO project, we found the percentage of structured inquiry laboratory experiments (level 0.5) dropped to 33.6% and the percentages of higher levels of inquiry (levels 1.5, 2, and 3) increased, as seen above in Figure 1.

From these data we can see that when faculty integrated some of the MICRO laboratory experiments into their course curriculum there was a shift from traditional, “cookbook” style laboratory experiments to laboratory experiments of higher levels of inquiry. We did not advocate for a complete removal of structured inquiry laboratory experiments (level 0.5) in the workshop but rather advocated for a more scaffolded approach where students are working toward higher levels of inquiry laboratory experiments. With these things in mind, the results were encouraging in the sense that faculty were providing students with more opportunities to engage in higher inquiry laboratory experiments.

We also determined the changes in levels of inquiry excluding the MICRO laboratory experiments implemented by each institution to see the extent to which faculty changed the rest of their laboratory curriculum. We observed that there was still a decrease in the percentage of structured inquiry laboratory experiments and an increase in the percentages of higher inquiry laboratory experiments as seen in Figure 1.

It was encouraging that faculty in the MICRO project made changes to their existing laboratory curriculum to incorporate some higher levels of inquiry laboratory experiments while also undertaking the implementation of inquiry-based MICRO laboratory experiments themselves. The experience of implementing some of the MICRO laboratory experiments was important in that it allowed faculty to get a feel for facilitating higher inquiry-based laboratory experiments and to see firsthand how student learning might be enhanced because of it. We examined each institution’s laboratory materials for changes in inquiry and while not everyone made drastic changes from their initial instructional practices, about half did make noticeable changes to their curriculum beyond the incorporation of the MICRO laboratory experiments. One individual went from having a laboratory curriculum of all cookbook (level 0.5) laboratory materials to having all level 2 laboratory experiments in addition to the MICRO laboratory experiments as they decided to drastically change the structure of their laboratory course to have students generate a procedure in groups ahead of lab. Two other individuals included final projects that were authentic inquiry (level 3) where students used the microfluidic techniques they learned through the MICRO laboratory experiments to approach a new research question. There was only one instance of an institution where the level of inquiry dropped from the preimplementation curriculum to the postimplementation curriculum and that was largely due to a change in modality from a very project-based curricula in person to a remote modality.

Along with examining each institution’s laboratory curriculum for changes in inquiry, we also checked to see if there were any definite trends in scaffolding such as by starting the semester with lower levels of inquiry and ending the semester with higher levels of inquiry. This was something we discussed in the workshops as a method of preparing students for higher inquiry experiences. There were some institutions whose curriculum fell into this approach, but that was not consistent across all. However, when a level 3, authentic inquiry laboratory experiment was present, it was always a project at the end of the semester.

**Figure 4:**
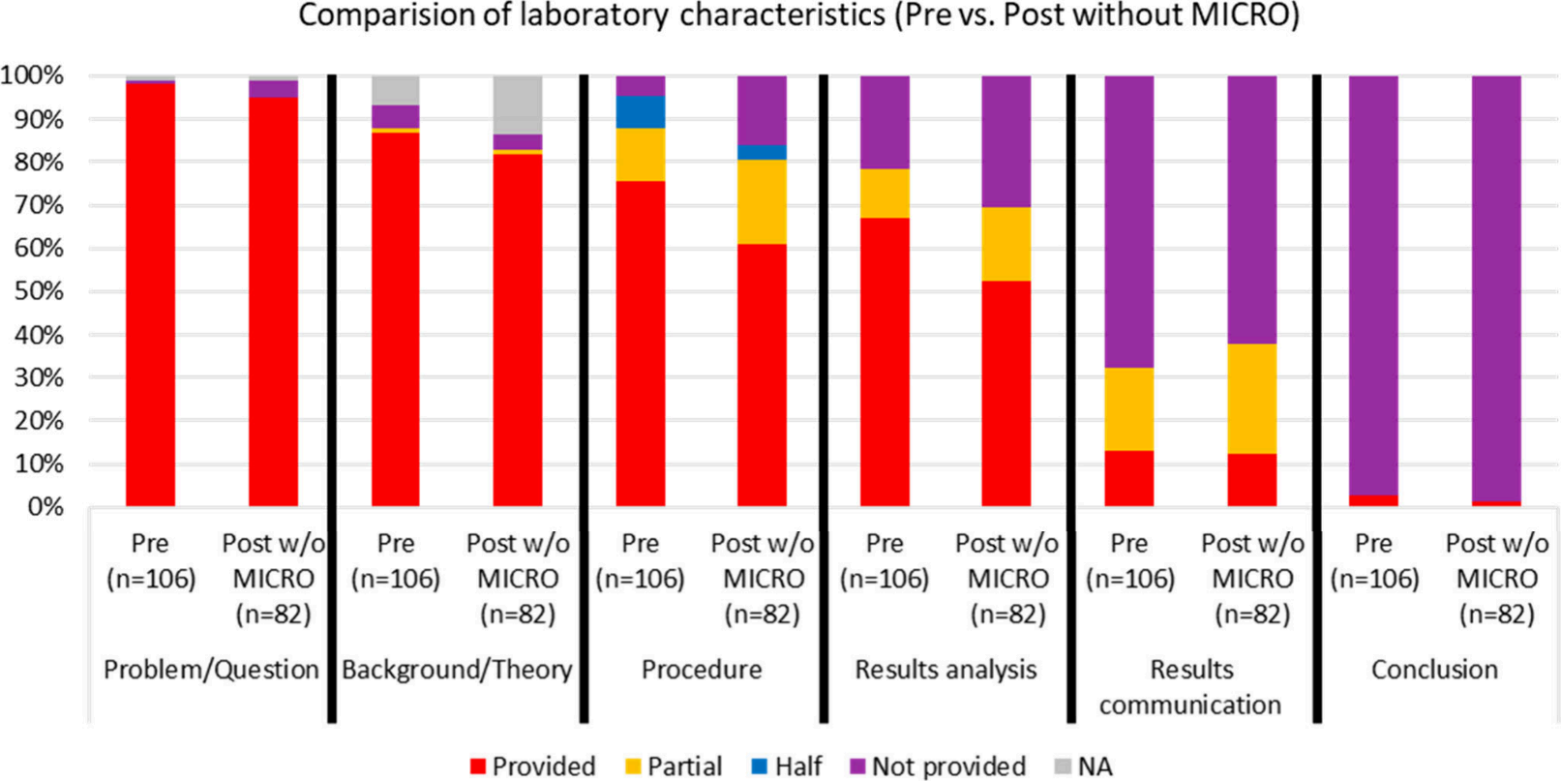
Results of the analysis of pre and post (without MICRO) laboratory materials broken down for each laboratory component.

To get a better understanding of how laboratory curricula shifted toward higher levels of inquiry, we investigated what laboratory characteristics were provided, partially provided, or not provided to students as seen in Figure 4.

There is little change in problem/question and theory/background, which are still largely provided to students. However, when looking at procedure/design and results analysis we can see some differences in what is provided to students and what students must generate. We see more partially provided and not provided for both the procedure/background and the results analysis when comparing the postimplementation (without MICRO) data to the preimplementation data. This indicates that faculty made changes to their existing curriculum to provide students with more opportunities to generate these aspects of the laboratory experiment. In the workshop we talked about strategies for modifying or designing laboratory experiments to be a next level higher in inquiry by reducing or eliminating what is provided to students for namely procedure/design and results analysis.

While 52.4% of the laboratory experiments were still structured inquiry (level 0.5), we recognize that there were several factors, such as personal and contextual factors, that influence faculty curricular decisions. One such factor was time. With faculty already preparing to implement two to four MICRO laboratory experiments that were new to them while managing normal course load and research demands, often while also navigating a new learning modality due to the pandemic, time was limited for modifying existing laboratory experiments. Additionally, in the workshops we discussed options for scaffolding a course in terms of level of inquiry where students may engage in a few lower inquiry laboratory experiments at the start of a course to build necessary skills for higher levels of inquiry, so we would not anticipate a complete disappearance of the lower-level laboratory experiments.

## Limitations

One limitation to this work is that faculty in the MICRO project applied to be a part of this project and may not be representative of all analytical chemistry faculty members. Particularly with cohort 2, we had a large fall off on response rate for surveys and material submission, limiting our ability to accurately represent that cohort. Since this study focused on the experiences of analytical chemistry faculty and their laboratory materials, there may be limited transferability to other disciplines of chemistry. Another limitation to this study is that we defined instructional practices as the level of inquiry of laboratory experiments used by participants. This is just one element of instructional practices. We did not conduct laboratory observations or collect other forms of data to give a more wholistic picture of participants’ instructional practices. Lastly, we were not able to obtain data from participants in semesters following their participation in the MICRO project to be able to assess retention of inquiry-based laboratory experiments.

## Implications

### Implications for Research

Through this research we expanded the levels of inquiry rubric designed by Bruck et al.^15^ to allow for the characterization of laboratory materials that fall between the discrete levels in the rubric. This modified rubric can be used by other researchers to characterize laboratory materials for other courses or to examine the impact of faculty development programs on adopting inquiry-based laboratory practices. A future direction for this work could be to follow faculty over time to see how they continue to modify their laboratory experiments to be more inquiry-based.

### Implications for Practitioners

Through this research there are several suggestions for practitioners who are looking to adopt a more inquiry-based approach for their laboratory courses. One suggestion is that practitioners who are interested in moving toward more inquiry-based approaches could benefit from applying the modified Bruck et al. rubric to their own laboratory curriculum. Doing so will identify which laboratory components are being provided to students and highlight ways to modify the laboratory experiments to be higher inquiry by removing laboratory elements partially or fully. Another suggestion for practitioners who want to design inquiry-based laboratory activities or modify their existing laboratory activities to be more inquiry-based would be to consider the goals that they have for students for the laboratory activity in terms of knowledge and skills. These goals should guide what inquiry-elements or components of the laboratory activity are provided to students or left up to them to generate. For instance, if the goal is to teach students a new technique, providing the laboratory procedure would be important, but if the goal is that students can choose an appropriate method for answering a research question, then having students generate a laboratory procedure would be important. Additionally, attention should be paid to not reducing the level of inquiry of a laboratory activity by providing more structure in places intentionally left open for student discovery, as we saw in a few instances in our analysis.

## Conclusion

The MICRO project was designed to support faculty in the adoption of inquiry-based laboratory experiments. We used a modified version of the inquiry rubric designed by Bruck et al.^15^ to assess the levels of inquiry of laboratory materials used by instructors prior to their involvement in the MICRO project and then the semester they implemented the MICRO laboratory experiments. We found that the laboratory experiments that each institution used prior to their involvement in the MICRO project were primarily structured inquiry (level 0.5) laboratory experiments, which match the findings of other studies.^15,31,61^ We saw a decrease in the number of structured inquiry laboratory experiments and an increase in laboratory experiments of higher levels of inquiry during the semester that faculty implemented the MICRO laboratory experiments. In addition to the incorporation of MICRO laboratory experiments into their curriculum, many faculty made changes to their existing curriculum to increase the level of inquiry of laboratory experiments. In particular, we saw more partially provided and not provided procedure and results analysis sections of the laboratory experiments. Findings from this work highlight how the inquiry rubric by Bruck et al. can be used to analyze and inform curricular changes to laboratory curriculum to increase its level of inquiry. A move toward more inquiry-based laboratory experiences will allow students to engage in the authentic practices of scientists and be equipped with the skills that they need to be successful in the workforce.
